# Does a deep seated L5 vertebra position with respect to the iliac crests affect the accuracy of percutaneous pedicle screw placement at lumbosacral junction?

**DOI:** 10.1186/s12891-017-1537-x

**Published:** 2017-05-05

**Authors:** Jing Guo, Lianjin Guo, Juzhou Gao, Qinjie Ling, Zhixun Yin, Erxing He

**Affiliations:** 1 Spine Surgery, The First Affiliated Hospital of Guangzhou Medical University, Guangzhou, China; 2 Guangzhou Orthopaedic Institute, Guangzhou, China; 3 The First Affiliated Hospital of Guangzhou Medical University, 151 Yanjiang West Road, 510000 Guangzhou, China

**Keywords:** Percutaneous pedicle screw, Accuracy, Lumbosacral junction, Minimally invasive spine surgery

## Abstract

**Background:**

Significant prominence of iliac crests with a deep seated L5 vertebra can potentially interfere with the screw trajectory when placing percutaneous pedicle screws (PPS) at the lumbosacral segment. The objective of this study was to investigate the influence of L5 position in relation to the iliac crests on the accuracy of percutaneous placement of lumbosacral pedicle screws.

**Methods:**

From Oct 2012 to Sep 2014, 54 patients who underwent PPS placement at L5-S1 segment were recruited. Patients were divided into 2 groups: the L5-Seated Group (L5-S Group, *n* = 34) including patients with intercrest lines passing through the L4 vertebra or L4/5 intervertebral disc; whereas the L5-Non-Seated Group (L5-NS Group, *n* = 20) including patients with intercrest lines passing through the L5 vertebra. Postoperative computerized tomography was obtained in all patients, and PPS accuracy was evaluated by grading pedicle breach (Grade 0, no breach; Grade 1, ≤2mm; Grade 2, >2mm without neurological compromise; Grade 3, with complications). Screw convergence angle (SCA), defined as the angle subtended by the screw axis and vertebral midline, was also recorded.

**Results:**

In the L5-S Group, 82.4% (56/68) screws were measured as Grade 0 at L5, and 66.2% (45/68) were Grade 0 at S1; meanwhile, in the L5-NS Group, 77.5% (31/40) and 75.0% (30/40) screws were Grade 0 at L5 and S1, respectively. Misplacement rate was numerically higher at S1 in the L5-S Group (*P* > 0.05). There were significantly more medial pedicle violations at S1 in the L5-S Group as compared to the L5-NS Group (25.0% *vs* 7.5%, *P* = 0.024). No statistical difference was found in L5 SCA between the 2 groups (L5-S Group 23.7° ± 7.4° *vs* L5-NS Group 23.4° ± 10.6°, *P* = 0.945); however, S1 SCA was significantly smaller in the L5-S Group (14.7° ± 5.8°) when compared with the L5-NS Group (20.8° ± 5.2°) (*P* = 0.036).

**Conclusions:**

A deep seated L5 vertebra with respect to the iliac crests might compromise the accuracy of PPS placement at S1 vertebra. Severe iliac prominence may interfere with the screw trajectory and limit the medial angulation of pedicle screw for percutaneous S1 fixation.

## Background

Percutaneous placement of pedicle screws was first introduced by Magerl in 1977 [1]. This technique was initially described for the management of spinal fractures and infections, and designed for application of external spinal fixation systems [1]. However, due to its undesired clinical outcome and low patients’ compliance to external fixation, PPS was almost abandoned for decades. In 2001, Foley et al. [2] described a new system that could percutaneously place pre-curved rods onto polyaxial pedicle screws, and completed the first case of percutaneous internal spinal fixation for degenerative lumbar diseases. After that, PPS techniques have rapidly improved and led to the expanded use of this fixation method in minimally invasive spine surgery [3].

PPS techniques use small skin incisions and transmuscular approaches with no need to extensively dissect the paraspinal musculature for screw insertion [4]. As compared to conventional open techniques, the advantages of percutaneously placing pedicle screws include less blood loss, minimal soft tissue trauma, reduced postoperative pain, and faster rehabilitation et al. [2, 5–8]. Over the past decade, many studies have investigated the safety and accuracy of PPS, with reported rates of screw misplacement from 6.2 to 14.3% [9–15]. In general, percutaneous placement of pedicle screws is no inferior to open spine surgery; however, there is a potential towards medial and inferior penetration due to its lack of direct visualization of the accurate entry point [14].

Lumbosacral junction is the most common level for PPS instrumentation in minimally invasive posterior lumbar fusion [4]. Although usually a safe procedure in skilled hands, percutaneous placement of lumbosacral pedicle screws can be technically demanding [16]. The screw heads can impinge on one another at the L5/S1 level due to the L5 and S1 pedicle angulations [3]. Knowledge of lumbosacral anatomy, interpretation of intraoperative imaging, and tactile feedback of bony landmarks are all critical for lumbosacral PPS insertion [17]. In our practice, we have noticed that, in some patients who have significant prominence of the iliac crests with a deep seated L5 vertebra, placement of lumbosacral PPS is quite difficult and suboptimal placement even occurs. Anatomic relationship between the L5 vertebra and iliac crests may be of some clinical importance in minimally invasive lumbosacral instrumentation. To our best knowledge, no study has ever addressed this issue before. The objective of this study was to investigate whether a deep seated L5 vertebra with respect to the iliac crests could affect the safety and accuracy of percutaneous placement of lumbosacral pedicle screws.

## Methods

### Subjects

This is a retrospective study approved by the Clinical Research Ethics Committee of our hospital. From October 2012 to September 2014, 54 consecutive patients (26 males and 28 females) who underwent minimally invasive single-level fusion surgeries at L5/S1 segment were recruited. Patients were diagnosed as lumbar disc herniation, lumbar spinal stenosis, degenerative or isthmic spondylolisthesis. Patients with infection, spinal tumor, trauma-related lesions and previous spine surgery were excluded. The age range of the patients was 33 to 68 years old (mean, 49.4 ± 12.0 yrs).

**Fig. 1:**
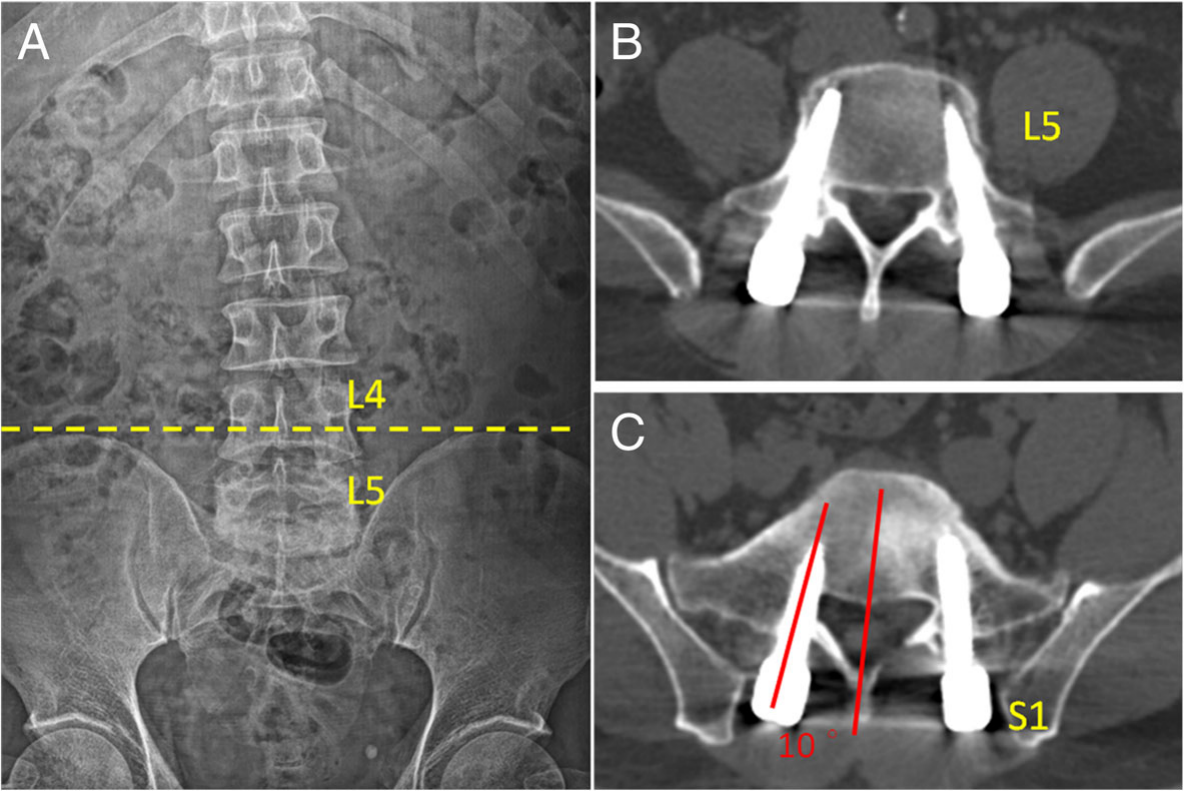
**a** Preoperative AP radiograph showing an intercrest line passing through the L4 vertebra (L5-Seated Group); **b** Postoperative axial CT image showing no pedicle breach (Grade 0 screw) at the L5 level; **c** Postoperative axial CT image showing a 5-mm medial pedicle breach of the right S1 screw leading to transient motor and sensory radiculopathy after surgery (Grade 3 screw)

**Fig. 2:**
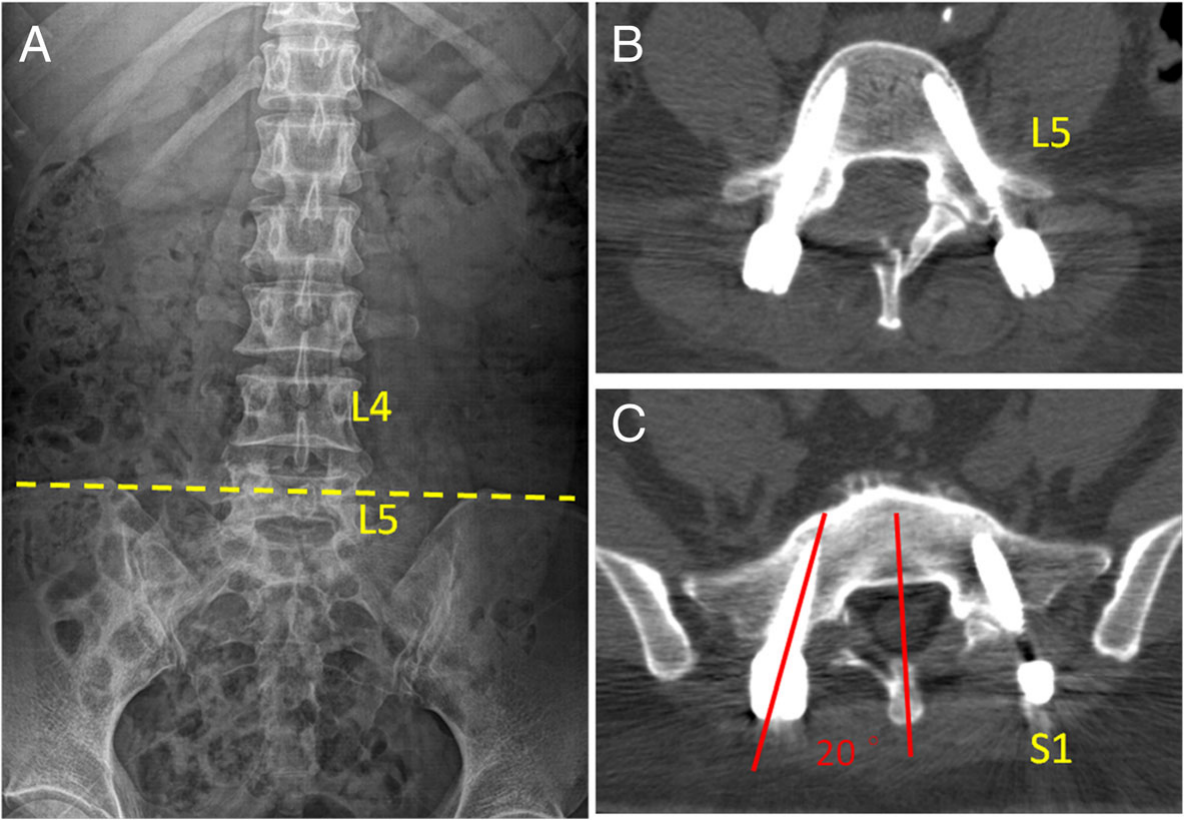
**a** Preoperative AP radiograph showing an intercrest line passing through the L5 vertebra (L5-Non-Seated Group); **b** and **c** Postoperative axial CT images showing no pedicle breach (Grade 0 screw) both at the L5 and S1 level

Patients were divided into 2 groups according to the position of L5 vertebra with respect to the iliac crests on anteroposterior (AP) radiographs of the lumbar spine. The L5-Seated Group (L5-S Group) included patients with intercrest lines passing through the L4 vertebra or L4/5 intervertebral disc (Fig. 1a); whereas, the L5-Non-Seated Group (L5-NS Group) included patients with intercrest lines passing through the L5 vertebra (Fig. 2a).

### Surgical procedure

All surgical procedures were performed by the same senior surgeon (EX. H.). A total of 216 screws were placed in 54 patients for percutaneous lumbosacral stabilization using the Luna Spine System (Double Medical, Xiamen, China). Percutaneous pedicle screws ranged in diameter from 6.0 mm to 7.0 mm and were inserted according to the technique described in previous literatures [4, 18, 19].

Briefly, under fluoroscopy, the lateral aspect of the pedicle was marked on the skin. A 1-cm stab incision was made lateral to the mark. Then, a Jamshidi needle was introduced and docked onto the lateral aspect of the pedicle, which was called the “3 o’clock” position. Lateral fluoroscopic view was obtained to adjust the cephalocaudal direction of the Jamshidi needle so that the needle shaft was parallel to the superior endplate. After that, the Jamshidi needle was advanced 20 mm to 25 mm into the pedicle until its tip reached the posterior border of the vertebral body. On the AP view, the needle tip should remain lateral to the medial pedicle wall, with no more than three-quarters across the pedicle. Gently advance the Jamshidi needle 10 mm to 15 mm more into the vertebral body, and therefore it was “safe” with no risk of medial pedicle breach. Afterwards, a Kirschner (K)-wire was placed down the barrel of the Jamshidi needle. Once a satisfactory penetration of the pedicle with the K-wire was achieved, the Jamshidi needle was removed. Tissue guards were then deployed over the K-wire to perform soft tissue dissection. A pedicle screw tap was placed down the trajectory of the K-wire. Finally, the appropriate pedicle screw was inserted.

### Evaluation of screw position

Postoperative computed tomography (CT) was obtained for all patients to assess the screw position using a 128-detector row CT unit (SOMATOM Definition AS, Siemens, USA). The CT examination was performed within the first week after surgery, with slices acquired in helical mode in a craniocaudal direction. The raw data were reconstructed in transverse 2-mm-thick slices with an increment of 1 mm for visualization of the lumbosacral spine. Sagittal and coronal reformats of the spine were obtained as well.

Two independent observers analyzed the digital CT scans of all instrumented pedicles, with both individual and consensus interpretations for each screw. Evaluation of screw position was performed according to the grading system published by Raley et al. [12], modified to include assessment of significant complications that were likely to require revision surgery. Screw accuracy was defined as Grade 0 (screw within the pedicle cortex), Grade 1 (screw thread breach pedicle wall ≤ 2 mm), Grade 2 (screw thread breach pedicle wall > 2 mm, but without neurological compromise), and Grade 3 (screw with complications: pedicle fracture, anterior breach with neurovascular compromise, lateral/medial breach with neurological sequelae).

In addition, the direction of pedicle breach was noted as well. The screw convergence angle (SCA) was measured for all pedicle screws, and defined as the angle subtended by a line parallel to the vertebral midline and a line through the axis of the screw tract (Figs. 1c and 2c).

### Statistical analysis

Differences between the L5-S Group and L5-NS Group were evaluated on the basis of gender, screw position grade, and breach direction using chi-square test and Fisher exact test. Independent-samples t test was performed for age, body mass index and screw convergence angle differences. A *P* value < 0.05 was considered to be significant. Statistical analysis was performed using a commercial software package SPSS 16.0 (SPSS, Chicago, IL, USA).

## Results

Patient demographics were shown in Table 1. Of the 54 patients with lumbosacral PPS fixation, 34 patients with 136 screws were in the L5-S Group and 20 patients with 80 screws were in the L5-NS Group. Mean age was 51.7 ± 12.7 years in the L5-S Group and 46.6 ± 11.8 years in the L5-NS Group (*P* = 0.514). The difference in gender composition was not significant between the 2 groups (*P* = 0.358). In the L5-S Group, 101 screws (74.3%, 101/136) were placed in the cortical shell of the pedicle (Grade 0), *versus* 61 screws (76.3%, 61/80) in the L5-NS Group. Regarding the severity of screw malposition, minor pedicle breach (Grade 1) was most common in both groups (L5-S Group, 18.4%; L5-NS Group, 17.5%). There was no significant difference in the distribution of screw position grade between the 2 groups (*P* = 0.886).

**Table 1:** Patient demographics and screw position

Variable	L5-S group	L5-NS group	*P*
Patient no.	34	20	-
Male (%)	18 (52.9%)	8 (40.0%)	0.358
Female (%)	16 (47.1%)	12 (60.0%)	
Mean age (y)	51.7 ± 12.7	46.6 ± 11.8	0.514
BMI (kg/m^2^)	25.7 ± 6.7	26.5 ± 7.1	0.482
Screw no.	136	80	-
Grade 0 (%)	101 (74.3%)	61 (76.3%)	0.886
Grade 1 (%)	25 (18.4%)	14 (17.5%)	
Grade 2 (%)	9 (6.6%)	5 (6.3%)	
Grade 3 (%)	1 (0.7%)	0 (0.0%)	

We further analyzed the screw accuracy with respect to the vertebral level in 2 groups (Table 2). At L5 level, 82.4% (56/68) screws were Grade 0 in the L5-S Group; and 77.5% (31/40) screws were Grade 0 in the L5-NS Group. The distribution of screw position grade was similar between the 2 groups at L5 level (*P* = 0.784). However, at S1 level, the percentage of Grade 0 screws was found to be numerically lower in the L5-S Group (66.2%, 45/68) compared to that in the L5-NS Group (75.0%, 30/40), although the difference did not reach statistical significance (*P* > 0.05). The L5-S Group had a relatively higher Grade 1 malposition rate at S1 level than the L5-NS Group (22.1% *versus* 17.5%). Only 1 Grade 3 pedicle violation was noted in the L5-S Group at S1 level. The patient had a 5-mm medial breach of the right S1 pedicle, and developed transient motor and sensory radiculopathy. The symptom resolved spontaneously at the 3-month follow-up after surgery.

**Table 2:** Evaluation of screw position according to vertebral level

	At L5			At S1		
Variable	L5-S group	L5-NS group	*P*	L5-S group	L5-NS group	*P*
Screw no.	68	40	-	68	40	-
Grade 0 (%)	56 (82.4%)	31 (77.5%)	0.784	45 (66.2%)	30 (75.0%)	0.720
Grade 1 (%)	10 (14.7%)	7 (17.5)		15 (22.1%)	7 (17.5%)	
Grade 2 (%)	2 (2.9%)	2 (5.0%)		7 (10.3)	3 (7.5%)	
Grade 3 (%)	0 (0.0%)	0 (0.0%)		1 (1.5%)	0 (0.0%)	
Pedicle breach	12	9	-	23	10	-
Lateral (%)	7 (10.3%)	6 (15.0%)	0.468	6 (8.8%)	7 (17.5%)	0.181
Medial (%)	5 (7.4%)	3 (7.5%)	0.978	17 (25.0%)	3 (7.5%)	0.024
Mean SCA (°)	23.7° ± 7.4°	23.4° ± 10.6°	0.945	14.7° ± 5.8°	20.8° ± 5.2°	0.036

As for the direction of pedicle breach, no significant difference was noted at L5 level between the 2 groups (*P* > 0.05). Meanwhile, at S1 level, there were more medial pedicle violations in the L5-S Group as compared to the L5-NS Group (25.0% *versus* 7.5%, *P* = 0.024). The mean SCA of L5 screws was 23.7° ± 7.4° in the L5-S Group *versus* 23.4° ± 10.6° in the L5-NS Group, without statistical significance (*P* = 0.945). However, the mean SCA of S1 screws was found to be significantly smaller in the L5-S Group (14.7° ± 5.8°) when compared with the L5-NS Group (20.8° ± 5.2°) (*P* = 0.036).

## Discussion

The current study attempted to investigate the influence of lumbosacral morphology in terms of L5 vertebra position with respect to the iliac crests on the accuracy of PPS placement. A total of 54 consecutive patients, divided into the L5-S and L5-NS Groups, were available for evaluation. Based on Table 1, patients of the 2 groups were very comparable, which formed a good baseline for comparison. Our results indicated that a deep seated L5 vertebra with respect to the iliac crests might compromise the accuracy of PPS placement at S1 level, particularly in limiting the convergent angulation of S1 pedicle screw.

Percutaneous techniques for posterior spinal instrumentation use small muscle-splitting approaches to allow placement of pedicle screws under fluoroscopic guidance [4]. These approaches permit minimally invasive screw insertion at multiple levels while avoiding the extensive soft tissue trauma associated with conventional open approaches [3]. Kim et al. [5] found that PPS fixation showed no significant decrease in the cross-sectional area of multifidus muscle after surgery against open technique, and had positive effects on postoperative trunk muscle performance. In addition, significant reduction of blood loss was achieved. Wang et al. [20] showed an average blood loss of 49.3 ± 34.0 ml *versus* the open approach needing 311.5 ± 246.4 ml for treatment of traumatic thoracolumbar fractures. A faster rehabilitation due to the less invasive technique has also been reported by different scholars [20–23].

The safety and accuracy of PPS placement remain significant concerns for surgeons, due to its lack of direct visualization and heavy dependence on intraoperative fluoroscopy. Nevertheless, favorable results have been reported in literatures. Ringel et al. [10] reviewed 104 patients treated with PPS fixation. Of 488 pedicle screws, 87% of screw positions were rated good, 10% were rated acceptable, and only 3% were rated unacceptable. Nine patients had revision surgery due to pedicle breaches. Raley et al. [12] analyzed 424 percutaneously inserted screws from T4 to S1. They found that 383 screws (90.3%) were placed in the cortical shell of the pedicle. Forty-one screws (9.7%) were misplaced, of which only 2 screws presented clinical complications. In a recent study, Smith et al. [15] reported a pedicle breach rate of 6.2% in 601 instrumented pedicles. There were 2 symptomatic breaches, both associated with a medial breach at the L5 pedicle.

Comparison of screw accuracy between the percutaneous and open techniques was also reported. Oh et al. [14] revealed that the pedicle breach rates were not statistically different between the percutaneous and open techniques. However, the open technique showed a relatively higher incidence of lateral breach, whereas medial, superior, and inferior breaches were higher for the percutaneous technique. In this lumbosacral junction-focused study, the overall acceptable rate of pedicle screws (Grade 0 and 1) was 93.1% (201/216). And, the majority of breaches (72.2%, 39/54) were relatively small (≤2 mm). Only 1 patient developed transient neurological deficit due to a 5-mm medial breach at the S1 pedicle. The results of the current study were comparable to those reported in previous literatures.

PPS insertion at the lumbosacral junction can be technically demanding. The sophisticated and variant regional anatomy can result in suboptimal screw placement. In our practice, we usually encountered difficulties in the placement of lumbosacral PPS for some patients who had a deep seated L5 position with respect to the iliac crests. We hypothesized that the anatomic relationship between the L5 vertebra and iliac crests may influence the accuracy of PPS placement at the lumbosacral junction. To the best of our knowledge, no report has described this phenomenon before.

In the current study, the accuracy of PPS at L5 level was similar between the L5-S and L5-NS Groups (Table 2) (Figs. 1b and 2b). However, for S1 pedicle screws, the L5-S Group had a relatively lower percentage of Grade 0 screws than the L5-NS Group, although without statistical significance (Table 2). Further analysis showed that there were more medial pedicle breaches in the L5-S Group as compared to the L5-NS Group at S1 level (Table 2). In addition, the mean SCA of S1 screws was 14.7° ± 5.8° in the L5-S Group, which was significantly smaller than that in the L5-NS Group (20.8° ± 2.3°) (*P* = 0.036) (Fig. 1c and 2c). Our results indicated that a deep seated L5 position with respect to the iliac crests might compromise the accuracy of PPS placement at S1 level. A significantly prominent ilium could affect the finding of the ideal entry point for S1 pedicle screw. The tip of the Jamshidi needle could be docked too medially due to the obstruction of iliac crests at the lateral side, which potentially leads to medial pedicle breach and small convergence angle. Therefore, in these patients, special attention and careful manipulation should be taken for percutaneously placement of pedicle screws at the lumbosacral junction.

There are some potential limitations in this study. First, although the data were collected from a single experienced spine surgeon, the universal involvement of residents and fellows in these cases may partially deviate the results. Second, evaluation of the superior and inferior pedicle breach was not included. The height of the L5 and S1 pedicle was quite large, very few cases of superior or inferior breach was noted in our series. Finally, the current study only utilized traditional C-arm fluoroscopy for PPS placement. Advancing techniques using computer-assisted 2D and 3D navigation systems may provide advantages to PPS placement at the lumbosacral junction.

## Conclusions

A deep seated L5 vertebra position with respect to the iliac crests might compromise the accuracy of PPS placement at S1 vertebra. Severe iliac prominence may interfere with the screw trajectory and affect the finding of the ideal entry point for S1 pedicle screw, potentially increasing the risk of medial pedicle breach during percutaneous S1 fixation.

## Abbreviations

AP: Anteroposterior
CT: Computed tomography
PPS: Percutaneous pedicle screw
SCA: Screw convergence angle
